# Duration of first admission and its relation to the readmission rate in a psychiatry hospital

**DOI:** 10.4103/0019-5545.58294

**Published:** 2009

**Authors:** S. Vasudeva, M. S. Narendra Kumar, K. Chandra Sekhar

**Affiliations:** Asha Hospital, 298, Road No.14, Banjara Hills, Hyderabad - 500 034, India

**Keywords:** First admission, readmission, psychiatry hospital

## Abstract

**Background:**

Most psychiatric illnesses have a chronic relapsing course. It is estimated that the readmission rate for discharged patients is approximately 40-50% within one year of their discharge from the hospital. The current emphasis in mental health service is on brief hospitalization and providing community-based services.

**Aim:**

To understand the relationship between the duration of first hospital admission and the rates of readmission in a psychiatry hospital.

**Materials and Methods:**

All the patients admitted at Asha hospital, Hyderabad for the first time between 16 September, 2003 to 15 March, 2004, were included in the study. The hospital records of these patients were examined and the data was collected on various variables, which included demographic variables, duration of hospital stay, diagnosis, and the number of readmissions for a period of approximately three-and-a-half years. The duration of the first hospital stay was divided into four categories, Group 1:1-7 days, Group 2:8 to 14 days, Group 3:15 to 30 days, and Group 4: More than 30 days.

**Results:**

The sample consisted of 516 patients, out of whom 17 were excluded because of insufficient data. Two hundred and fifty patients belonged to Group 1 (1 to 7 days), 206 patients in Group 2 (8 to 14 days), Group 3 (15 to 30 days) constituted 35 patients, and eight patients were in Group 4 (>30 days).

**Conclusion:**

The length of the initial hospital stay is important to prevent future hospitalization. There are no definite predictors for readmission that could be detected, except for the length of the initial admission in the study.

## INTRODUCTION

Most of the psychiatric illnesses have a chronic relapsing course. It is estimated that the readmission rate for discharged patients is approximately 40-50% within one year of their discharge from hospital.[1] The current emphasis in mental health service is on brief hospitalization and providing community-based services. In India, apart from the family support system there is very little community-care support provided by the mental health care service providers, both in the public and the private sectors. About 50% of all patients admitted to the psychiatry hospitals are readmissions. Patients who are admitted for longer durations have had appropriate treatment planning, follow-up, and lesser readmission after discharge.[2] There is a great likelihood that brief stay patients will be rehospitalized within 30 days after discharge rather than patients treated for longer periods.[3] An increase in the length of stay from 9 to 26 days was associated with a 55% reduction in the rate of rapid readmission.[4] The patients with above-average length of stay were rarely readmitted and most of the readmissions returned during the first year after discharge.[5] The study by Lieberman *et al*., showed that the length of stay of the patient had no impact on the improvement of depressive symptoms in depressed patients.[6] Patients with greater impairment in self-care, more severe symptoms, and a more persistent illness were more likely to be readmitted than other patients.[7] Premature discharge was not associated with readmission rates.[7,8] Studies done on readmission rates in American and European psychiatric hospitals, provided information both in favor of brief hospitalization and longer hospitalization and the results seem equivocal. Our literature search did not yield much information on this aspect in the Indian context.

### Aim of the study

To understand the relationship between the duration of the first hospital admission and the rate of readmission in a psychiatry hospital.

### Hypothesis

Duration of admission, although dependent upon many variables, in this study, the consultant's decision to discharge patients was dependent on the immediate relief of the patient symptoms, to the extent that it was manageable. However, it was felt that if the first hospital stay, (index admission) was prolonged, then the patient got a better insight into the illness and the chances of readmission were minimal. We hypothesized that when the duration of the first hospitalization was short, the rates of readmission were higher and when the duration of the first hospitalization was short, the time for rehospitalization was also short.

## MATERIALS AND METHODS

All the patients admitted at Asha hospital, Hyderabad, for the first time between 16 September 2003 and 15 March 2004, were included in the study [Flowchart 1]. The hospital records of these patients were studied and the data was collected on various variables, which included demographic variables, duration of hospital stay, diagnosis, and the number of readmissions for a period of approximately three-and-a-half years. The duration of the first hospital stay was divided into four categories, Group 1:1-7 days, Group 2:8 to 14 days, Group 3:15 to 30 days, and Group 4: More than 30 days.

**Flowchart 1:**
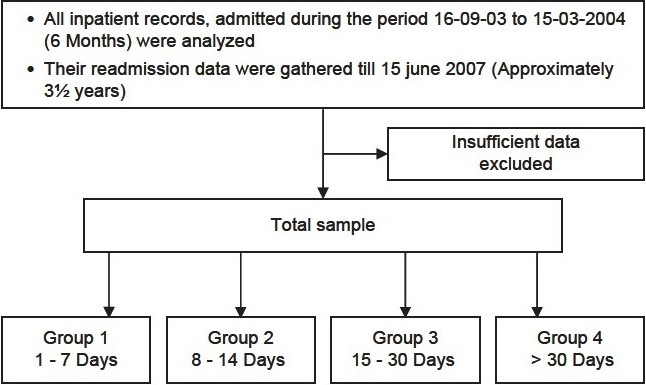
Methodology

### Statistical analysis

Description of data is done depicting the percentage and the bar diagrams. Test of comparison is done for continuous variables using the ‘*t*’ test, and for discrete variables, the Chi-square test.

## RESULTS

The sample consisted of 516 patients, out of which 17 were excluded because of insufficient data available from the records [Flowchart 2]. Two hundred and fifty patients belonged to Group 1 (1 to 7 days), 206 patients to Group 2 (8 to 14 days), Group 3 (15 to 30 days) constituted 35 patients, and there were eight patients in Group 4 (>30 days).

**Flowchart 2:**
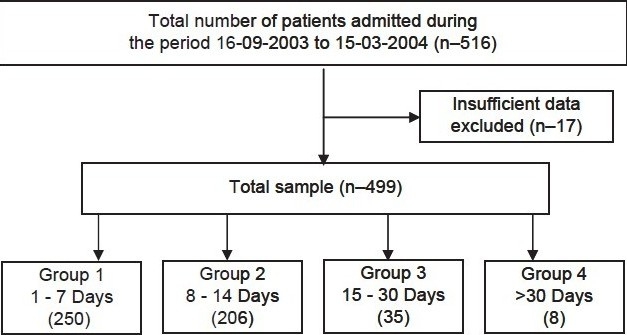
Result

Table 1 shows the sociodemographic profile. The mean age across the groups were in the range of 33.62 to 36.06 years, indicating that a majority of them were in the early thirties. Male sex constituted the majority of the sample (69.2 to 85.8%). More than two-thirds of the patients were from an urban background and 60-80% lived in a nuclear family. Interestingly Group 4 showed the least percentage of marriage, with 37% and maximum divorces of 12.5% and this was statistically significant (*P* < 0.05). Graduates were a majority across all groups ranging from 40 to 60%. A majority of them were unemployed, at 20 to 40%, and unskilled workers were few in number (3-6%), which was statistically significant (*P* < 0.05).

**Table 1:** Sociodemographic profile

Variables	Group 1 (n-250) (%)	Group 2 (n-206) (%)	Group 3 (n-35) (%)	Group 4 (n-8) (%)	df	*P*
Mean age	33.62 ± 13.68	33.33 ± 14.83	35.06 ± 13.54	34.37 ± 13.63	1	NS
Gender						
Male	173 (69.2)	158 (76.7)	30 (85.8)	6 (75.0)	3	NS
Female	77 (30.8)	48 (23.3)	5 (14.2)	2 (25.0)	3	NS
Education					15	NS
Illiterate	5 (2)	2 (0.9)	00	00		
Primary	24 (9.6)	10 (4.8)	1 (2.8)	1 (12.5)		
Secondary	49 (19.6)	32 (15.5)	4 (11.4)	00		
Intermediate	70 (38.8)	60 (29.1)	10 (28.5)	2 (25)		
Graduate	97 (38.8)	95 (46)	20 (60)	4 (50)		
Post graduate	8 (3.2)	7 (3.3)	00	1 (12.5)		
Locality					3	NS
Rural	51 (20.4)	49 (23.7)	5 (14.2)	2 (25)		
Urban	199 (79.6)	157 (76.3)	30 (85.8)	8 (100)		
Marital status					6	0.001
Single	95 (38)	103 (50)	19 (54.2)	4 (50)		
Married	153 (61.2)	100 (48.5)	15 (42.8)	3 (37.5)		
Divorced	2 (0.8)	3 (1.45)	1 (2.8)	1 (12.5)		
Type of family					3	NS
Nuclear	171 (68.4)	144 (70)	29 (82.9)	5 (62.5)		
Extended	79 (31.6)	62 (30)	6 (17.1)	3 (37.5)		
Occupation					36	0.01
Student	41 (16.4)	23 (11.1)	2 (5.7)	1 (12.5)		
Business	46 (18.4)	28 (13.5)	6 (17.1)	3 (37.5)		
Unemployed	53 (21.2)	80 (38.8)	15 (42.8)	2 (25		
Agriculture	20 (8)	14 (6.7)	1 (2.8)	00		
Professional	11 (4.4)	3 (1.45)	1 (2.8)	00		
Service	18 (7.2)	20 (9.7	4 (11.4)	1 (12.5)		
Unskilled work	16 (6.4)	7 (3.3	00	00		
Housewife	45 (18)	32 (15.5)	5 (14.2)	1 (12.5)		

NS: Not significant

**Figure 1:**
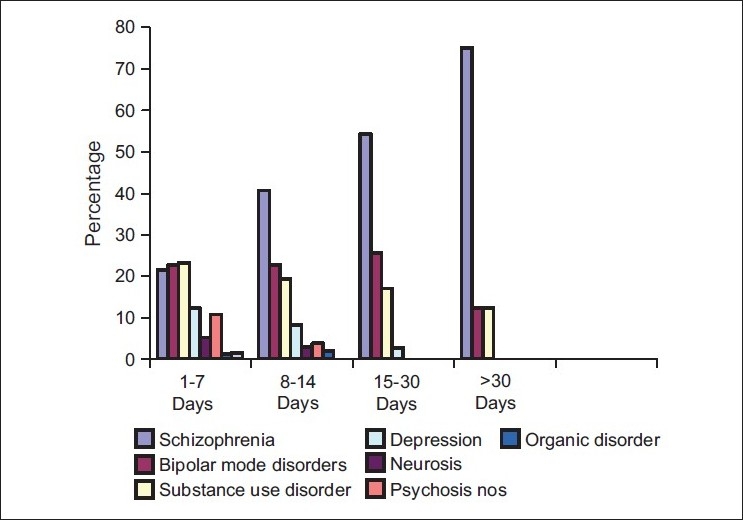
Diagnosis across all groups during first admission

Figure 1 Bar chart shows diagnosis across the entire groups during the first admission. Schizophrenia predominates across the entire group with 21% in Group 1 and 75% in Group 4. Approximately 22% of the patients in Groups 1, 2, and 3 had bipolar disorder. The percentage of substance use disorder declined across the group from 23% in Group 1 to 12% in Group 4.

Overall, 25 to 30% of the patients were readmitted since their first admission [Table 2]. About 20% of the patients in the first three groups had one readmission. The total percentage of ≥2 admissions was 14 and 11% in Group 1 and Group 2, respectively, whereas, only 5.7% of the patients in Group 3 readmitted more than twice during the study period, which is statistically significant across the group (*P* = 0.001).

**Table 2:** Number of readmission across the group

Number of readmission	1-7 days (Group 1: n-250) (%)	8-14 days (Group 2: n-206) (%)	15-30 days (Group 3: n-35) (%)	>30 days (Group 4: n-8) (%)
1	44 (17.6)	39 (18.9)	7 (20)	00
2	19 (7.6)	16 (7.76)	1 (2.85)	1 (12.5)
3	3 (1.21)	3 (1.45)	1 (2.85)	1 (12.5)
4	4 (1.6)	2 (0.97)	00	00
>5	9 (3.6)	2 (0.97)	00	00
Total	79 (31.6)	62 (30.05)	9 (25.7)	2 (25)

*P* = 0.001

**Figure 2:**
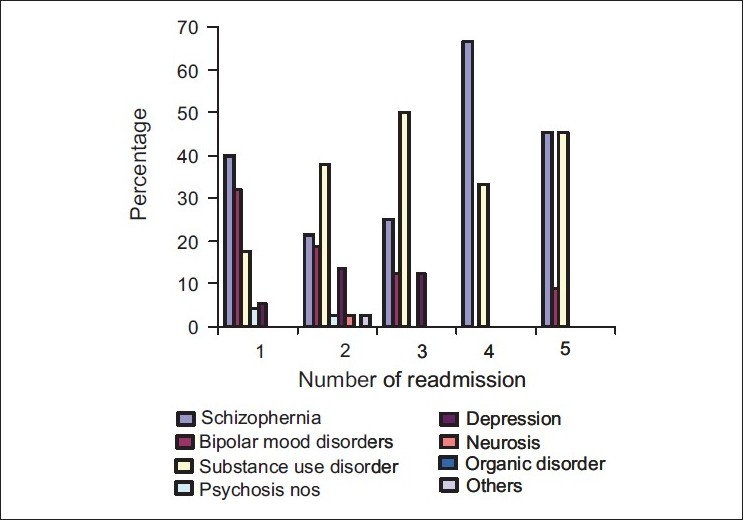
Diagnosis across readmission

Figure 2 shows the diagnosis across the number of readmissions. Schizophrenia and substance use disorder accounted for more than 60% of the diagnosis in all the readmissions. Thirty to fifty percent of the patients who were readmitted more than twice had substance use disorder. Approximately 67% of the patients who had four readmissions had schizophrenia.

**Figure 3:**
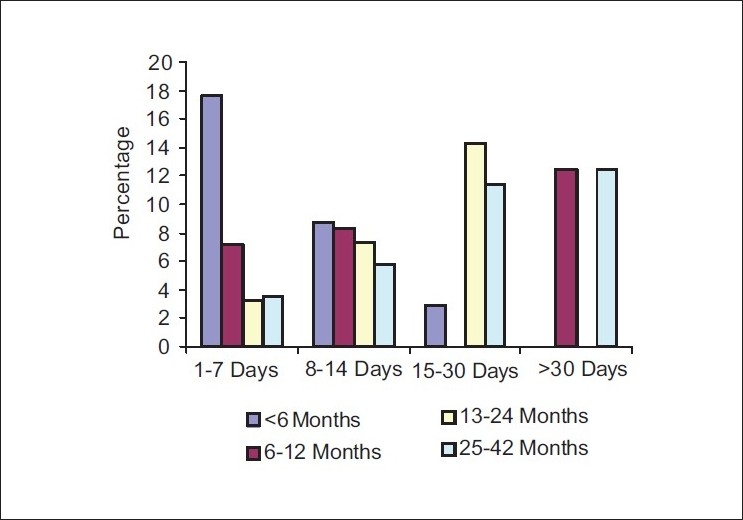
Time to relapse since first admission

Figure 3 shows the time to relapse since the first admission. The percentage of patients who were readmitted within the first six months after initial discharge was the highest, with 17% in Group 1, whereas, only 3% were readmitted in Group 3. Approximately 25% of the patients in Group 3 were readmitted after one year and almost the same trend was seen in Group 4 as well.

## DISCUSSION

The result of this study indicates that readmission occurs earlier when the length of the first admission is short. About 50% of the readmissions occur within the first six months, when the length of the first admission is short (1-7 days), and readmission occurs after 1-2 years or even more when the length of the initial hospitalization is longer (>2 weeks). This finding is in conformity with the results of studies across the American and European hospitals. Brief hospitalizations of less than nine days led to rapid readmissions, within one month.[4] The three groups showed no significant differences as to amount of improvement in levels of psychopathology at 3 and 12 weeks, but the briefly hospitalized patients were able to resume their vocational roles sooner. Increase in the length of stay from nine days to 26 days was associated with 55% reduction in the readmissions.[4] While patients with above average length of stay in the hospital were rarely readmitted, a majority returned within one year and nearly all had returned within the three-year period.[5] Patients with brief hospital stay were likely to get readmitted within 30 days of discharge as compared to those who stayed for longer periods.[3]

In this study, the reason for lesser readmissions in patients with longer initial stay can be attributed to the following factors:

1. With longer hospital stay, at the time of discharge there is near total remission of symptoms.
2. Patients gain better insight into their illness.
3. Follow-up treatment is better.
4. Psychoeducation to the patient and family members is adequately performed and discharge planning is better.

This study did not count the previous admissions, as very little data was available with regard to those. However, this should not be influencing the hypothesis as the current admission was taken as the index admission and rehospitalization was calculated after the index admission.

The hospital being a private one has a good follow-up program and medication compliance is reasonably adequate. Rehospitaliszation after the index admission, whenever it occurred, had been recorded, which was taken into account while determining the number of rehospitalizations

The patient's compliance with medication could not be ascertained from the available data and hence it was not mentioned. However, the hospital being a private one has a good follow-up program and medication compliance is reasonably adequate.

In this study, the percentage of divorces was as high as 12.5% in patients who got admitted for more than 30 days during initial hospitalization. This shows that this group of patients lacked adequate family support and hence compliance to the treatment might have been poor.

The present data shows that more than 60% of the cases who were readmitted had the diagnosis of schizophrenia and substance use disorder, and the number of readmissions were more for patients with substance use disorder. Readmission rates in substance use disorder, which mainly comprised of alcohol dependence, could be because of the fact that the hospital mainly catered to detoxification, and after detoxification the emphasis was on outpatient counseling.

Longer hospitalization was only on the basis of the treating doctor's evaluation and decision with regard to improvement of symptoms and not related to any social variables.

## CONCLUSION

The results of our study indicate that the length of initial hospital stay is important to prevent future hospitalization. Twenty-five to thirty percent of the patients get readmitted. There are no definite predictors for readmission that can be detected in the study, except for the length of initial admission.

### Limitations

- Retrospective data.
- Study confined to the variables in the data registry.
- Absence of clinical ratings.
- Previous admission details in other hospitals not considered elsewhere.
